# Supplementary material for: HIVprotI: an integrated web based platform for prediction and design of HIV proteins inhibitors
Source: J Cheminform. 2018 Mar 9;10:12. doi: 10.1186/s13321-018-0266-y (PMC5845081; doi:10.1186/s13321-018-0266-y)
Supplement: Supplementary file 2 — Additional file 2. Source code of HIVProtI web server. [file 13321_2018_266_MOESM2_ESM.zip › HIVprotI_Source-code/hivprotI/weka-3-6-6/doc/weka/core/AbstractStringDistanceFunction.html]

AbstractStringDistanceFunction


---


|  |  |  |
| --- | --- | --- |
| **Overview** \| **Package** \| **Class** \| **Tree** \| **Deprecated** \| **Index** \| **Help** \| **Weka's home** | |  |
| PREV CLASS   **NEXT CLASS** | **FRAMES**    **NO FRAMES**     **All Classes** |  |
| SUMMARY: NESTED \| FIELD \| CONSTR \| METHOD | DETAIL: FIELD \| CONSTR \| METHOD |  |


---


## weka.core Class AbstractStringDistanceFunction

```
java.lang.Object
  weka.core.NormalizableDistance
      weka.core.AbstractStringDistanceFunction
```

**All Implemented Interfaces:**: java.io.Serializable, DistanceFunction, OptionHandler, RevisionHandler

**Direct Known Subclasses:**: EditDistance

---

``` public abstract class AbstractStringDistanceFunction extends NormalizableDistance ```

Represents the abstract ancestor for string-based distance functions, like
EditDistance.

**Version:**
:   $Revision: 1.1 $

**Author:**
:   Bruno Woltzenlogel Paleo

**See Also:**: Serialized Form

---

**Field Summary**

**Fields inherited from class weka.core.NormalizableDistance**

`R_MAX, R_MIN, R_WIDTH`


**Constructor Summary**

`AbstractStringDistanceFunction()`
  
          Constructor that doesn't set the data

`AbstractStringDistanceFunction(Instances data)`
  
          Constructor that sets the data


| **Method Summary** | |
| --- | --- |
| `double` | `distance(Instance first, Instance second, double cutOffValue, PerformanceStats stats)`             Calculates the distance between two instances. |

**Methods inherited from class weka.core.NormalizableDistance**

`attributeIndicesTipText, distance, distance, distance, dontNormalizeTipText, getAttributeIndices, getDontNormalize, getInstances, getInvertSelection, getOptions, getRanges, globalInfo, initializeRanges, initializeRanges, initializeRanges, initializeRangesEmpty, inRanges, invertSelectionTipText, listOptions, postProcessDistances, rangesSet, setAttributeIndices, setDontNormalize, setInstances, setInvertSelection, setOptions, toString, update, updateRanges, updateRanges, updateRanges, updateRangesFirst`

**Methods inherited from class java.lang.Object**

`equals, getClass, hashCode, notify, notifyAll, wait, wait, wait`

**Methods inherited from interface weka.core.RevisionHandler**

`getRevision`

**Constructor Detail**

### AbstractStringDistanceFunction

```
public AbstractStringDistanceFunction()
```

:   Constructor that doesn't set the data

---


### AbstractStringDistanceFunction

```
public AbstractStringDistanceFunction(Instances data)
```

:   Constructor that sets the data

    **Parameters:**: `data` - the set of instances that will be used for later distance comparisons


**Method Detail**

### distance

```
public double distance(Instance first,
                       Instance second,
                       double cutOffValue,
                       PerformanceStats stats)
```

:   Calculates the distance between two instances. Offers speed up (if the
    distance function class in use supports it) in nearest neighbour search by
    taking into account the cutOff or maximum distance. Depending on the
    distance function class, post processing of the distances by
    postProcessDistances(double []) may be required if this function is used.

    :   **Specified by:**: `distance` in interface `DistanceFunction` **Overrides:**: `distance` in class `NormalizableDistance`
    :   **Parameters:**: `first` - the first instance: `second` - the second instance: `cutOffValue` - If the distance being calculated becomes larger than cutOffValue then the rest of the calculation is discarded.: `stats` - the performance stats object **Returns:**: the distance between the two given instances or Double.POSITIVE\_INFINITY if the distance being calculated becomes larger than cutOffValue.


---


|  |  |  |
| --- | --- | --- |
| **Overview** \| **Package** \| **Class** \| **Tree** \| **Deprecated** \| **Index** \| **Help** \| **Weka's home** | |  |
| PREV CLASS   **NEXT CLASS** | **FRAMES**    **NO FRAMES**     **All Classes** |  |
| SUMMARY: NESTED \| FIELD \| CONSTR \| METHOD | DETAIL: FIELD \| CONSTR \| METHOD |  |


---
